# *N*-[(*E*)-(9-Ethyl-9*H*-carbazol-3-yl)methyl­idene]aniline

**DOI:** 10.1107/S1600536810018660

**Published:** 2010-05-26

**Authors:** Nuray Yeksan, Ece Uzkara, Orhan Zeybek, Erol Asker

**Affiliations:** a Department of Physics, Faculty of Arts and Sciences, Balıkesir University, 10615 Cağış–Balıkesir, Turkey; b Necatibey Faculty of Education, Balıkesir University, 10100 Balıkesir, Turkey

## Abstract

The title compound, C_21_H_18_N_2_, was obtained as the product of the reaction between 9-ethyl-9*H*-carbazole-3-carbaldehyde and aniline in ethanol. The crystal packing is stabilized mainly by C—H⋯π inter­actions between the carbazole benzene rings and the methyl­ene H atoms.

## Related literature

For background to photoconductive properties see: Segura (1998 ▶); Grigoras & Antonoaia (2005 ▶). For geometrical parameters in related structures, see: Wang *et al.* (2008 ▶); Huang *et al.* (2008 ▶). 
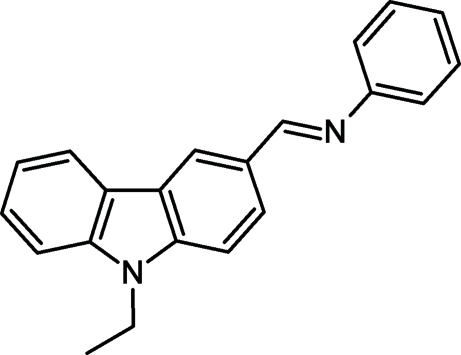


## Experimental

### 

#### Crystal data

C_21_H_18_N_2_
*M* *_r_* = 298.37
Monoclinic, 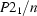

*a* = 15.3350 (3) Å
*b* = 5.9692 (10) Å
*c* = 17.5447 (3) Å
β = 91.162 (1)°
*V* = 1605.7 (3) Å^3^
*Z* = 4
Mo *K*α radiation
μ = 0.07 mm^−1^
*T* = 295 K
0.6 × 0.4 × 0.2 mm

#### Data collection

Rigaku R-AXIS RAPID S diffractometer
28963 measured reflections
2838 independent reflections
2821 reflections with *I* > 2σ(*I*)
*R*_int_ = 0.030

#### Refinement

*R*[*F* ^2^ > 2σ(*F* ^2^)] = 0.062
*wR*(*F* ^2^) = 0.148
*S* = 1.41
2838 reflections
209 parameters
H-atom parameters constrained
Δρ_max_ = 0.14 e Å^−3^
Δρ_min_ = −0.13 e Å^−3^

### 

Data collection: *CrystalStructure* (Rigaku & Rigaku/MSC, 2003 ▶); cell refinement: *CrystalStructure*; data reduction: *SORTAV* (Blessing, 1995 ▶); program(s) used to solve structure: *SIR92* (Altomare *et al.*, 1993 ▶); program(s) used to refine structure: *SHELXL97* (Sheldrick, 2008 ▶); molecular graphics: *ORTEP-3 for Windows* (Farrugia, 1997 ▶); software used to prepare material for publication: *WinGX* (Farrugia, 1999 ▶).

## Supplementary Material

Crystal structure: contains datablocks global, I. DOI: 10.1107/S1600536810018660/om2337sup1.cif

Structure factors: contains datablocks I. DOI: 10.1107/S1600536810018660/om2337Isup2.hkl

Additional supplementary materials:  crystallographic information; 3D view; checkCIF report

## Figures and Tables

**Table 1:** Hydrogen-bond geometry (Å, °) *Cg*1 and *Cg*2 are the centroids of the C1–C4/C4*A*/C9*A* and C4*B*/C5–C8/C8*A* rings, respectively.

*D*—H⋯*A*	*D*—H	H⋯*A*	*D*⋯*A*	*D*—H⋯*A*
C5—H5⋯*Cg*1^i^	0.93	2.87	3.587 (3)	135
C12—H12⋯*Cg*2^i^	0.93	2.98	3.660 (3)	131
C10—H10*A*⋯*Cg*2^ii^	0.97	3.25	4.050 (4)	142

Symmetry codes: (i) 
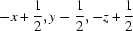
; (ii) 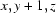.

**Fig. 1.**
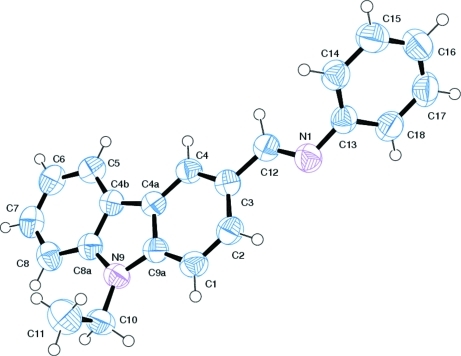
The structure of the title compound with the atom numbering scheme. The displacement ellipsoids are drawn at the 50% probability level and arbitrary spheres are shown for the H atoms.

**Fig. 2.**
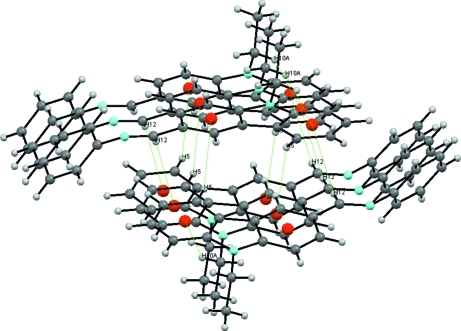
Packing diagram of showing C—H···Cg intermolecular interactions; red spheres represent ring centroids.

## supplementary crystallographic information

### Comment

The structure of the title compound is depicted in (Fig. 1). The bond lengths and internal bond angles of the carbazole skeleton are comparable to those of related molecules (Wang *et al.*, 2008; Huang *et al.*, 2008). The carbazole and phenyl skeletons are essentially planar with r.m.s deviations of 0.021Å (carbazole ring) and 0.008Å (phenyl ring). The phenyl ring is twisted away from the carbazole ring by 67.45 (05)°. The ethyl group protrudes out of the plane of the carbazole skeleton as indicated by the C9A—N9—C10—C11 torsion angle of 86.0 (3)°. The only force that stack the molecules appears to be π-ring C—H···Cg intermolecular interactions among the benzene rings of carbazole and the hydrogen atoms H5, H10A and H12 (Fig. 2).

### Experimental

The title compound was synthesized via the imine reaction between aniline and 9-ethyl-9*H*-carbazol-3-carbaldehyde in ethanol. In a round bottom flask fitted with a magnetic stirrer a solution was prepared from 9-ethyl-9*H*-carbazol-3-carbaldehyde (1.116 g, 5 mmol) and aniline (0.70 g, 7.5 mmol) in 50 ml ethanol at ambient temperature. After stirring for 2 h, the solution was left for crystallization overnight, after which time the product was precipitated as yellow crystals. The crude product was separated by filtration and washed with ethanol. Yellow, transparent crystals suitable for the X-ray diffraction analysis were grown from tetrahydrofuran by slow evaporation technique at ambient temperature, mp. 407 K. FT—IR (KBr) ν_max_ (cm^-1^): 3048 (Ar—H), 2973 (-CH_3_), 2930 (-CH_2_-), 1618 (C=N), 1587, 1567 (Ar—N), 1489, 1473, 1461 (Ar C=C); ^1^HNMR (300 MHz, CDCl_3_, ppm): 1.46 (t, J = 7.3 Hz, 3H, CH_3_), 4.38 (q, J = 7.3 Hz, 2H, -CH_2_-), 7.21-7.59 (m, 9H, ArH), 8.07 (dd, J = 8.5 and 1.8 Hz, 1H, H2), 8.18 (dt, J = 7.9 and 0.8 Hz, 1H, H5), 8.64 (s, 1H, H12), 8.65 (d, J= 1.8, 1H, H4). UV-Vis, [EtOH, λmax (nm), (ε)] = 238 (25800), 293 (22100), 338 (18500).

### Refinement

All non-hydrogen atoms were refined anisotropically; the hydrogen atoms were positioned geometrically and allowed to ride on their corresponding parent atoms with C—H distances of 0.93Å (aromatic), 0.96Å (methyl), and 0.97Å (methylene) with *U*_iso_(H) =1.5*U*_eq_(C) of the parent atom for the methyl group and 1.2*U*_eq_(C) for the rest.

### Crystal data

**Table:**

C_21_H_18_N_2_	*F*(000) = 632
*M**_r_* = 298.37	*D*_x_ = 1.234 Mg m^−^^3^
Monoclinic, *P*2_1_/*n*	Mo *K*α radiation, λ = 0.71073 Å
Hall symbol: -P 2yn	Cell parameters from 8828 reflections
*a* = 15.3350 (3) Å	θ = 2.3–25.3°
*b* = 5.9692 (10) Å	µ = 0.07 mm^−^^1^
*c* = 17.5447 (3) Å	*T* = 295 K
β = 91.162 (1)°	Prism, yellow
*V* = 1605.7 (3) Å^3^	0.6 × 0.4 × 0.2 mm
*Z* = 4	

### Data collection

**Table:**

Rigaku R-AXIS RAPID S diffractometer	*R*_int_ = 0.030
graphite	θ_max_ = 25.2°, θ_min_ = 2.3°
ω scans	*h* = −18→18
28963 measured reflections	*k* = −6→7
2838 independent reflections	*l* = −20→20
2821 reflections with *I* > 2σ(*I*)	

### Refinement

**Table:**

Refinement on *F*^2^	Secondary atom site location: difference Fourier map
Least-squares matrix: full	Hydrogen site location: inferred from neighbouring sites
*R*[*F*^2^ > 2σ(*F*^2^)] = 0.062	H-atom parameters constrained
*wR*(*F*^2^) = 0.148	*w* = 1/[σ^2^(*F*_o_^2^) + (0.0417*P*)^2^ + 0.4831*P*] where *P* = (*F*_o_^2^ + 2*F*_c_^2^)/3
*S* = 1.41	(Δ/σ)_max_ = 0.002
2838 reflections	Δρ_max_ = 0.14 e Å^−^^3^
209 parameters	Δρ_min_ = −0.12 e Å^−^^3^
0 restraints	Extinction correction: *SHELXL97* (Sheldrick, 2008)
Primary atom site location: structure-invariant direct methods	Extinction coefficient: 0.0123 (17)

### Special details

**Table:**

Geometry. All s.u.'s (except the s.u. in the dihedral angle between two l.s. planes) are estimated using the full covariance matrix. The cell s.u.'s are taken into account individually in the estimation of s.u.'s in distances, angles and torsion angles; correlations between s.u.'s in cell parameters are only used when they are defined by crystal symmetry. An approximate (isotropic) treatment of cell s.u.'s is used for estimating s.u.'s involving l.s. planes.
Refinement. Refinement of *F*^2^ against ALL reflections. The weighted *R*-factor wR and goodness of fit *S* are based on *F*^2^, conventional *R*-factors *R* are based on *F*, with *F* set to zero for negative *F*^2^. The threshold expression of *F*^2^ > 2σ(*F*^2^) is used only for calculating *R*-factors(gt) etc. and is not relevant to the choice of reflections for refinement. *R*-factors based on *F*^2^ are statistically about twice as large as those based on *F*, and *R*- factors based on ALL data will be even larger.

### Fractional atomic coordinates and isotropic or equivalent isotropic displacement parameters (Å^2^)

**Table:**

	*x*	*y*	*z*	*U*_iso_*/*U*_eq_	
N1	0.53644 (12)	−0.2326 (3)	0.39241 (11)	0.0627 (5)	
N9	0.15252 (12)	0.1918 (3)	0.39179 (10)	0.0567 (5)	
C1	0.31091 (15)	0.1662 (4)	0.42835 (13)	0.0583 (6)	
H1	0.3127	0.2991	0.456	0.07*	
C2	0.38396 (14)	0.0359 (4)	0.42111 (12)	0.0580 (6)	
H2	0.436	0.0829	0.444	0.07*	
C3	0.38256 (14)	−0.1669 (4)	0.38000 (12)	0.0530 (5)	
C4	0.30554 (14)	−0.2380 (4)	0.34500 (12)	0.0531 (5)	
H4	0.304	−0.3717	0.3178	0.064*	
C4A	0.23047 (13)	−0.1080 (4)	0.35070 (11)	0.0511 (5)	
C4B	0.14167 (13)	−0.1318 (4)	0.32328 (11)	0.0520 (5)	
C5	0.09773 (15)	−0.2925 (4)	0.27965 (12)	0.0611 (6)	
H5	0.1274	−0.4154	0.2605	0.073*	
C6	0.00979 (16)	−0.2669 (5)	0.26525 (14)	0.0678 (7)	
H6	−0.0202	−0.3737	0.2364	0.081*	
C7	−0.03468 (16)	−0.0827 (5)	0.29346 (14)	0.0684 (7)	
H7	−0.0942	−0.0697	0.2833	0.082*	
C8	0.00672 (15)	0.0809 (4)	0.33593 (13)	0.0636 (6)	
H8	−0.0235	0.2044	0.354	0.076*	
C8A	0.09549 (14)	0.0541 (4)	0.35072 (11)	0.0542 (5)	
C9A	0.23398 (14)	0.0931 (4)	0.39289 (11)	0.0522 (5)	
C10	0.12624 (16)	0.3717 (4)	0.44220 (14)	0.0651 (6)	
H10A	0.0808	0.4594	0.4171	0.078*	
H10B	0.1757	0.4697	0.452	0.078*	
C11	0.0931 (2)	0.2849 (5)	0.51707 (15)	0.0874 (9)	
H11A	0.0766	0.4088	0.5486	0.131*	
H11B	0.1382	0.2003	0.5425	0.131*	
H11C	0.0434	0.1904	0.5077	0.131*	
C12	0.46110 (14)	−0.3017 (4)	0.37283 (12)	0.0553 (5)	
H12	0.4557	−0.4456	0.3529	0.066*	
C13	0.60811 (14)	−0.3801 (4)	0.38491 (12)	0.0550 (5)	
C14	0.61128 (15)	−0.5881 (4)	0.41922 (13)	0.0636 (6)	
H14	0.5646	−0.6363	0.448	0.076*	
C15	0.68271 (18)	−0.7249 (5)	0.41133 (16)	0.0767 (7)	
H15	0.6844	−0.8639	0.4352	0.092*	
C16	0.75146 (18)	−0.6566 (5)	0.36830 (18)	0.0818 (8)	
H16	0.7992	−0.7505	0.362	0.098*	
C17	0.74958 (16)	−0.4498 (5)	0.33467 (16)	0.0795 (8)	
H17	0.7964	−0.4032	0.3057	0.095*	
C18	0.67877 (15)	−0.3096 (4)	0.34333 (14)	0.0674 (6)	
H18	0.6786	−0.1679	0.3212	0.081*	

### Atomic displacement parameters (Å^2^)

**Table:**

	*U*^11^	*U*^22^	*U*^33^	*U*^12^	*U*^13^	*U*^23^
N1	0.0557 (11)	0.0652 (12)	0.0671 (12)	0.0002 (9)	−0.0018 (9)	−0.0068 (10)
N9	0.0579 (11)	0.0550 (11)	0.0573 (10)	0.0052 (9)	0.0051 (8)	−0.0043 (9)
C1	0.0646 (13)	0.0538 (13)	0.0566 (12)	−0.0033 (11)	0.0010 (10)	−0.0071 (10)
C2	0.0570 (12)	0.0616 (14)	0.0553 (12)	−0.0056 (11)	−0.0028 (10)	−0.0016 (11)
C3	0.0549 (12)	0.0564 (13)	0.0477 (11)	0.0000 (10)	0.0020 (9)	0.0005 (10)
C4	0.0592 (12)	0.0516 (12)	0.0485 (11)	−0.0004 (10)	0.0014 (9)	−0.0044 (9)
C4A	0.0549 (12)	0.0514 (12)	0.0470 (11)	−0.0026 (10)	0.0012 (9)	0.0000 (9)
C4B	0.0558 (12)	0.0567 (12)	0.0435 (10)	−0.0015 (10)	0.0022 (9)	0.0047 (9)
C5	0.0686 (14)	0.0626 (14)	0.0519 (12)	−0.0042 (11)	−0.0041 (10)	−0.0013 (11)
C6	0.0647 (14)	0.0795 (17)	0.0589 (13)	−0.0129 (13)	−0.0095 (11)	0.0054 (12)
C7	0.0551 (13)	0.0889 (19)	0.0611 (14)	−0.0045 (13)	−0.0032 (11)	0.0162 (13)
C8	0.0590 (13)	0.0738 (16)	0.0582 (13)	0.0065 (12)	0.0053 (10)	0.0093 (12)
C8A	0.0573 (12)	0.0593 (13)	0.0461 (11)	−0.0012 (10)	0.0037 (9)	0.0062 (10)
C9A	0.0569 (12)	0.0518 (12)	0.0480 (11)	0.0003 (10)	0.0051 (9)	0.0009 (9)
C10	0.0702 (15)	0.0567 (13)	0.0688 (15)	0.0077 (12)	0.0092 (12)	−0.0066 (11)
C11	0.101 (2)	0.102 (2)	0.0603 (15)	0.0029 (18)	0.0132 (14)	−0.0073 (15)
C12	0.0577 (13)	0.0579 (13)	0.0505 (12)	−0.0021 (10)	0.0031 (9)	−0.0029 (10)
C13	0.0519 (12)	0.0608 (13)	0.0524 (12)	−0.0049 (10)	−0.0024 (9)	−0.0068 (10)
C14	0.0626 (14)	0.0665 (15)	0.0619 (14)	−0.0044 (12)	0.0076 (11)	−0.0003 (11)
C15	0.0802 (17)	0.0668 (16)	0.0833 (18)	0.0074 (14)	0.0044 (14)	0.0030 (14)
C16	0.0649 (16)	0.082 (2)	0.099 (2)	0.0120 (14)	0.0043 (14)	−0.0125 (17)
C17	0.0560 (14)	0.093 (2)	0.0902 (19)	−0.0115 (14)	0.0155 (13)	−0.0126 (16)
C18	0.0640 (14)	0.0665 (15)	0.0720 (15)	−0.0111 (12)	0.0052 (12)	−0.0017 (12)

### Geometric parameters (Å, °)

**Table:**

N1—C12	1.268 (3)	C7—C8	1.376 (3)
N1—C13	1.416 (3)	C7—H7	0.93
N9—C9A	1.381 (3)	C8—C8A	1.390 (3)
N9—C8A	1.391 (3)	C8—H8	0.93
N9—C10	1.453 (3)	C10—C11	1.510 (3)
C1—C2	1.372 (3)	C10—H10A	0.97
C1—C9A	1.393 (3)	C10—H10B	0.97
C1—H1	0.93	C11—H11A	0.96
C2—C3	1.409 (3)	C11—H11B	0.96
C2—H2	0.93	C11—H11C	0.96
C3—C4	1.386 (3)	C12—H12	0.93
C3—C12	1.456 (3)	C13—C14	1.380 (3)
C4—C4A	1.393 (3)	C13—C18	1.384 (3)
C4—H4	0.93	C14—C15	1.375 (3)
C4A—C9A	1.411 (3)	C14—H14	0.93
C4A—C4B	1.442 (3)	C15—C16	1.371 (4)
C4B—C5	1.393 (3)	C15—H15	0.93
C4B—C8A	1.407 (3)	C16—C17	1.368 (4)
C5—C6	1.376 (3)	C16—H16	0.93
C5—H5	0.93	C17—C18	1.382 (4)
C6—C7	1.390 (4)	C17—H17	0.93
C6—H6	0.93	C18—H18	0.93
			
C12—N1—C13	118.5 (2)	N9—C9A—C1	129.1 (2)
C9A—N9—C8A	108.32 (18)	N9—C9A—C4A	109.32 (18)
C9A—N9—C10	124.65 (19)	C1—C9A—C4A	121.6 (2)
C8A—N9—C10	124.95 (19)	N9—C10—C11	112.2 (2)
C2—C1—C9A	117.8 (2)	N9—C10—H10A	109.2
C2—C1—H1	121.1	C11—C10—H10A	109.2
C9A—C1—H1	121.1	N9—C10—H10B	109.2
C1—C2—C3	122.0 (2)	C11—C10—H10B	109.2
C1—C2—H2	119	H10A—C10—H10B	107.9
C3—C2—H2	119	C10—C11—H11A	109.5
C4—C3—C2	119.6 (2)	C10—C11—H11B	109.5
C4—C3—C12	119.4 (2)	H11A—C11—H11B	109.5
C2—C3—C12	121.0 (2)	C10—C11—H11C	109.5
C3—C4—C4A	119.7 (2)	H11A—C11—H11C	109.5
C3—C4—H4	120.1	H11B—C11—H11C	109.5
C4A—C4—H4	120.1	N1—C12—C3	123.2 (2)
C4—C4A—C9A	119.24 (19)	N1—C12—H12	118.4
C4—C4A—C4B	134.2 (2)	C3—C12—H12	118.4
C9A—C4A—C4B	106.53 (18)	C14—C13—C18	118.8 (2)
C5—C4B—C8A	119.4 (2)	C14—C13—N1	122.6 (2)
C5—C4B—C4A	134.0 (2)	C18—C13—N1	118.5 (2)
C8A—C4B—C4A	106.66 (19)	C15—C14—C13	120.7 (2)
C6—C5—C4B	119.1 (2)	C15—C14—H14	119.6
C6—C5—H5	120.5	C13—C14—H14	119.6
C4B—C5—H5	120.5	C16—C15—C14	120.1 (3)
C5—C6—C7	120.6 (2)	C16—C15—H15	119.9
C5—C6—H6	119.7	C14—C15—H15	119.9
C7—C6—H6	119.7	C17—C16—C15	119.7 (3)
C8—C7—C6	122.0 (2)	C17—C16—H16	120.1
C8—C7—H7	119	C15—C16—H16	120.1
C6—C7—H7	119	C16—C17—C18	120.6 (2)
C7—C8—C8A	117.3 (2)	C16—C17—H17	119.7
C7—C8—H8	121.3	C18—C17—H17	119.7
C8A—C8—H8	121.3	C17—C18—C13	119.9 (2)
N9—C8A—C8	129.2 (2)	C17—C18—H18	120
N9—C8A—C4B	109.14 (18)	C13—C18—H18	120
C8—C8A—C4B	121.7 (2)		
			
C9A—C1—C2—C3	−0.6 (3)	C8A—N9—C9A—C1	−178.4 (2)
C1—C2—C3—C4	0.5 (3)	C10—N9—C9A—C1	−14.2 (4)
C1—C2—C3—C12	179.7 (2)	C8A—N9—C9A—C4A	1.4 (2)
C2—C3—C4—C4A	0.1 (3)	C10—N9—C9A—C4A	165.58 (19)
C12—C3—C4—C4A	−179.05 (19)	C2—C1—C9A—N9	179.7 (2)
C3—C4—C4A—C9A	−0.7 (3)	C2—C1—C9A—C4A	0.0 (3)
C3—C4—C4A—C4B	−178.9 (2)	C4—C4A—C9A—N9	−179.11 (19)
C4—C4A—C4B—C5	−1.2 (4)	C4B—C4A—C9A—N9	−0.5 (2)
C9A—C4A—C4B—C5	−179.6 (2)	C4—C4A—C9A—C1	0.7 (3)
C4—C4A—C4B—C8A	177.8 (2)	C4B—C4A—C9A—C1	179.34 (19)
C9A—C4A—C4B—C8A	−0.6 (2)	C9A—N9—C10—C11	−86.0 (3)
C8A—C4B—C5—C6	−0.8 (3)	C8A—N9—C10—C11	75.7 (3)
C4A—C4B—C5—C6	178.0 (2)	C13—N1—C12—C3	178.29 (19)
C4B—C5—C6—C7	0.3 (3)	C4—C3—C12—N1	168.4 (2)
C5—C6—C7—C8	0.5 (4)	C2—C3—C12—N1	−10.7 (3)
C6—C7—C8—C8A	−0.8 (3)	C12—N1—C13—C14	−56.6 (3)
C9A—N9—C8A—C8	178.3 (2)	C12—N1—C13—C18	125.4 (2)
C10—N9—C8A—C8	14.1 (4)	C18—C13—C14—C15	−1.0 (3)
C9A—N9—C8A—C4B	−1.7 (2)	N1—C13—C14—C15	−179.1 (2)
C10—N9—C8A—C4B	−165.92 (19)	C13—C14—C15—C16	−0.8 (4)
C7—C8—C8A—N9	−179.8 (2)	C14—C15—C16—C17	1.5 (4)
C7—C8—C8A—C4B	0.3 (3)	C15—C16—C17—C18	−0.4 (4)
C5—C4B—C8A—N9	−179.41 (18)	C16—C17—C18—C13	−1.5 (4)
C4A—C4B—C8A—N9	1.4 (2)	C14—C13—C18—C17	2.1 (3)
C5—C4B—C8A—C8	0.5 (3)	N1—C13—C18—C17	−179.7 (2)
C4A—C4B—C8A—C8	−178.60 (19)		

### Hydrogen-bond geometry (Å, °)

**Table:**

Cg1 and Cg2 are the centroids of the C1–C4/C4A/C9A and C4B/C5–C8/C8A rings, respectively.

**Table:**

*D*—H···*A*	*D*—H	H···*A*	*D*···*A*	*D*—H···*A*
C5—H5···Cg1^i^	0.93	2.87	3.587 (3)	135
C12—H12···Cg2^i^	0.93	2.98	3.660 (3)	131
C10—H10A···Cg2^ii^	0.97	3.25	4.050 (4)	142

Symmetry codes: (i) −*x*+1/2, *y*−1/2, −*z*+1/2; (ii) *x*, *y*+1, *z*.
